# Neural correlates of stress-reactive rumination in depression – The role of childhood trauma and social anxiety

**DOI:** 10.1016/j.ynstr.2024.100640

**Published:** 2024-05-09

**Authors:** David Rosenbaum, Isabell Int-Veen, Hendrik Laicher, Leonie Woloszyn, Ariane Wiegand, Sandra Ladegast, Ute Eßer, Agnes Kroczek, Daniel Sippel, Sebastian Menkor, Glenn Lawyer, Francesco Albasini, Christian Frischholz, Rainald Mössner, Vanessa Nieratschker, Elisabeth J. Leehr, Julian Rubel, Andreas J. Fallgatter, Ann-Christine Ehlis

**Affiliations:** a Department of Psychiatry and Psychotherapy, Tübingen Center for Mental Health (TüCMH), University Hospital of Tuebingen, Tuebingen, Germany; b Max Planck Institute of Psychiatry, Munich, Germany; c Machine Learning Solutions, Luxembourg; d Central Institute of Mental Health (CIMH), Mannheim, Germany; e German Center for Mental Health (DZPG), Germany; f Institute for Translational Psychiatry, University of Muenster, Germany; g Psychotherapy Research Lab, Psychology and Sport Sciences, Justus-Liebig-University Giessen, Giessen, Germany; h Dept. of Psychiatry and Psychotherapy, Central Institute of Mental Health, Medical Faculty Mannheim, University of Heidelberg, Germany

**Keywords:** Functional near-infrared spectroscopy, fNIRS, Rumination, Repetitive negative thinking, Perseverative thinking, Trier social stress test, Stress, Childhood trauma, Emotional abuse, Social anxiety, Cognitive control network, CCN, DLPFC

## Abstract

Recent work showed an association of prefrontal dysfunctions in patients with Major Depressive Disorder (MDD) and social stress induced rumination. However, up to date it is unclear which etiological features of MDD might cause prefrontal dysfunctions. In the study at hand, we aimed to replicate recent findings, that showed prefrontal activation alterations during the Trier Social Stress Test (TSST) and subsequently increased stress-reactive rumination in MDD compared to healthy controls. Moreover, we aimed to explore the role of adverse childhood experiences and other clinical variables in this relationship. N = 55 patients currently suffering from MDD and n = 42 healthy controls (HC) underwent the TSST, while cortical activity in areas of the Cognitive Control Network (CCN) was measured via functional near-infrared spectroscopy (fNIRS). The TSST successfully induced a stress reaction (physiologically, as well as indicated by subjective stress ratings) and state rumination in all subjects with moderate to large effect sizes. In comparison to HC, MDD patients showed elevated levels of state rumination with large effect sizes, as well as a typical pattern of reduced cortical oxygenation during stress in the CCN with moderate effect sizes. Self-reported emotional abuse and social anxiety were moderately positively associated with increased stress-reactive rumination. Within the MDD sample, emotional abuse was negatively and social anxiety positively associated with cortical oxygenation within the CCN with moderate to large effect sizes. In conclusion, our results replicate previous findings on MDD-associated prefrontal hypoactivity during stress and extends the research toward specific subtypes of depression.

## 1 Introduction

During the last two decades, research has emphasized the role of rumination and other types of repetitive negative thinking (RNT) in psychopathologies, such as Major Depressive Disorder (MDD). Rumination is common in MDD and has been consistently associated with increased symptom severity, worse treatment response, higher risk for relapse and increased risk for suicide (Bean et al., 2020; Smith and Alloy, 2009).

Although rumination is defined as a primary cognitive process, stressful emotional triggers have been shown to induce state rumination in observational as well as experimental studies (Connolly and Alloy, 2017; Pulopulos et al., 2022; Ruscio et al., 2015), with social interactions (e.g. arguments) and internal experiences (e.g. thoughts) being among the most prominent causes of rumination in MDD (Rosenbaum et al., 2022). Neural correlates of this stress-reactive rumination process include dysregulated functional connectivity between subcortical limbic areas such as the amygdala and prefrontal “cognitive” control areas such as the ventrolateral prefrontal cortex (VLPFC) (Fowler et al., 2017; Jacobs et al., 2014). During stress paradigms, such as the Trier Social Stress Test (Kirschbaum et al., 1993), Scan*STRESS* (Henze et al., 2020; Speicher et al., 2023) or the Montreal Imaging Stress Task (Dedovic et al., 2005), prefrontal hypoactivation is typically found in subjects with MDD and subjects prone to ruminate (Ming et al., 2017; Rosenbaum et al., 2018b, 2021). Additionally, meta-analytic data (Groenewold et al., 2013) suggest that during emotional processing MDD patients exhibit increased brain activity when exposed to negative content but decreased activity when exposed to positive stimuli in various subcortical brain regions including the amygdala, parahippocampus, striatum, cerebellum, fusiform, and anterior cingulate cortex. Conversely, within cortical areas, reduced activity in the dorsolateral prefrontal cortex (DLPFC) in response to negative stimuli, but increased activity in response to positive material is observed (Groenewold et al., 2013; Siegle et al., 2002). Emphasizing this data, it is suggested that depressed individuals exhibit heightened emotional reactivity towards negative stimuli, marked by hyperactivity within the limbic system, and hypoactivity in regions of the Cognitive Control Network (CCN) to regulate this emotionally charged process. Although the definitions of brain networks are not perfectly consistent between authors and studies (Witt et al., 2021), the CCN is roughly defined as a cortical network of mostly fronto-parietal regions involved in the implementation of cognitive control in various contexts, including the DLPFC, inferior frontal junction, anterior insular cortex and posterior parietal cortex (Breukelaar et al., 2016; Cole and Schneider, 2007; Rosenbaum et al., 2016). Recent studies have emphasized the involvement of the CCN in emotion regulation processes (Rosenbaum et al., 2020b, 2020c), the learning of psychotherapeutic techniques (Laicher et al., 2023; Rosenbaum et al., 2018, 2020d) and adaption to stress (De Wandel et al., 2023; Rosenbaum et al., 2018a; Thai et al., 2021), suggesting a pivotal role of this brain network in stress-reactive rumination (Int-Veen et al., 2023; Rosenbaum et al., 2018b, 2021; Witte et al., 2020), which seems to have a detrimental role in the regulation of emotional stress reactions (Laicher et al., 2023; Rosenbaum et al., 2022).

Etiological models of MDD emphasize the role of adverse life events in general and maltreatment during childhood in particular, besides genetic factors (Gibb et al., 2001, 2003; Nanni et al., 2012). Importantly, childhood maltreatment – as well as RNT (Ehring and Watkins, 2008) – is not only related to MDD, but also to other mental disorders such as anxiety disorders (Gardner et al., 2019) and psychosis (DeRosse et al., 2014). Notably, numerous reviews showed consistent associations of prefrontal dysfunction with childhood maltreatment (Bellis et al., 1999; Grassi-Oliveira and Stein, 2008; Weber and Reynolds, 2004) as well as anxiety disorders (Ball et al., 2013; Kenwood et al., 2022) such as social anxiety (Laicher et al., 2022), extending the hypothesized MDD-related association of RNT and prefrontal dysfunction towards etiological factors (maltreatment) as well as other mental disorders (e.g. anxiety disorders).

Leveraging the available evidence, we aimed to replicate the previous findings and explore the role of different clinical characteristics (e.g. childhood trauma, social anxiety, reported emotion regulation strategies, number of past depressive episodes) in stress-reactive rumination and prefrontal dysfunctions in MDD. On this account, in the present study we challenged patients with MDD and healthy controls (HC) with the TSST while using fNIRS to assess cortical blood oxygenation during the stress paradigm. Various clinical variables may be of interest (e.g. symptom severity, number of depressive episodes, trait rumination); here, we additionally focused on childhood trauma (Wiegand et al., 2021) and social anxiety (Laicher et al., 2022) with respect to their impact on stress-reactive rumination and its neural correlates in an exploratory analysis. Encompassing our objectives, we endeavored to address the following research questions:

Can we replicate previous findings that MDD subjects show increased stress-reactive rumination and decreased prefrontal activation during stress? What is the influence of clinical variables such as childhood maltreatment and social anxiety on the MDD-related effects?

We hypothesized that we could replicate our previous findings in the current investigation. More specifically, we expected the MDD group to show higher levels of subjective stress, negative affect and state rumination in comparison to HC, as well as higher reactivity in negative affect and state rumination. On a neural level, we expected a pattern of reduced cortical oxygenation in areas of the CCN under stress in the MDD group as compared to HC. We further expected that the effects of stress induction on prefrontal functioning and stress-reactive rumination would be enhanced in the MDD group having experienced trauma as compared to the MDD group without trauma. In the same way, we expected that the MDD group with social anxiety would show stronger stress-related effects in state rumination and prefrontal hypoactivity.

## 2 Methods

*Participants.* In total, 55 patients with a current diagnosis of a depressive disorder (MDD, 56.7 %), and 42 participants without any psychiatric diagnosis (HC, 43.3 %) were recruited via university circular emails and handing out flyers to local psychotherapists. All participants were screened using the Structured Clinical Interview for DSM-5 (SCID) (First et al., 2015). MDD patients were diagnosed by a trained psychologist/psychotherapist. Further, participants were asked if a diagnosis had been made by any other practitioner. HC were only included in case of no lifetime history of mental health disorders and no suspected disorders according to the SCID screening. Exclusion criteria for all participants further included acute physical illness, neurological disorders, acute substance abuse, and chronic or acute diseases that affect brain functioning (e.g. diabetes, kidney failure, cardiac diseases). All participants were aged 18–60 years and fluent in German. The ethics committee at the University Hospital and University of Tuebingen approved this project and all procedures are in line with the ethical standards of the Declaration of Helsinki. All participants gave their written informed consent prior to the participation in the study. On average, participants were 30.30 years old (*SD* = 10.69 years) and 73.20 % of the sample were female (see Table 1). 88% of the MDD sample were diagnosed with an F32.X diagnosis (depressive episode) and 12% with an F33.X diagnosis (recurrent depressive episode). Comorbid diagnoses were present in 51% of the cases: anxiety disorders (e.g. specific phobia) (40%), eating disorder (11%), and personality disorder (5%).

**Table 1:** Demographic data (means, standard deviations and *t*-/ $\chi^{2}$-tests) dependent on group (MDD = Major Depressive Disorder, HC = Healthy Controls).

Variable	HC (*n* = 42)	MDD (*n* = 55)	test statistic	*p*-value
age in years	27.29 (9.39)	32.60 (11.12)	*t*(93.99) = 2.55	<0.01
percent female	78.57 %	69.09 %	${\chi \left(1\right)}^{2}$ = 1.09	0.296
BDI-II total score	3.95 (4.58)	26.62 (7.29)	*t*(91.92) = 18.73	<0.001
RRS mean	1.57 (0.37)	2.77 (0.44)	*t*(94) = 14.40	<0.001
CTQ total score	32.04 (7.06)	44.44 (14.93)	*t*(79.38) = 5.38	<0.001
CTQ emotional abuse	6.80 (2.38)	11.53 (5.26)	*t*(77.72) = 5.90	<0.001
CTQ emotional neglect	7.93 (3.60)	11.98 (4.96)	*t*(93.61) = 4.63	<0.001
CTQ physical abuse	5.50 (1.33)	6.43 (3.06)	*t*(76.11) = 1.99	<0.05
CTQ physical neglect	6.45 (2.11)	7.74 (2.62)	*t*(93.87) = 2.66	<0.01
CTQ sexual abuse	5.37 (1.05)	6.77 (3.44)	*t*(65.32) = 2.83	<0.01
LSAS total score	21.10 (12.31)	56.80 (23.37)	*t*(83.77) = 9.63	<0.001

*Note.* Please note that CTQ and RRS data was missing in *n* = 1 MDD patient. BDI-II = Beck’s Depression Inventory II, RRS = Ruminative Response Scale, CTQ = Childhood Trauma Questionnaire (see supplemental material), LSAS = Liebowitz Social Anxiety Scale.

**Fig. 1:**
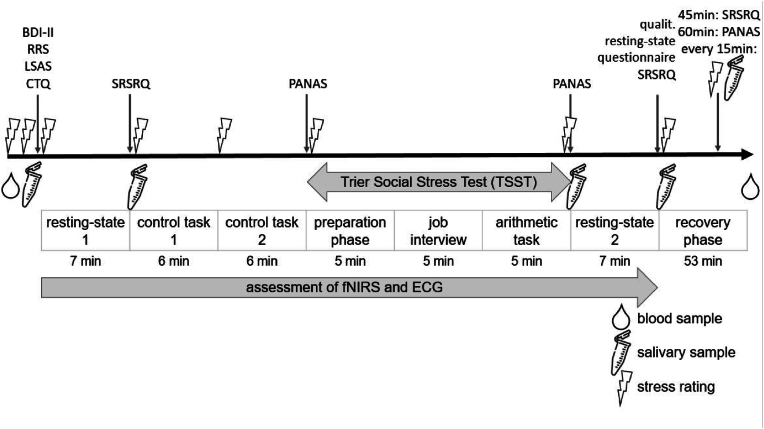
Experimental time course of the study. BDI-II = Beck Depression Inventory II, fNIRS = functional near-infrared spectroscopy, ECG = electrocardiography, RRS = Ruminative Response Scale, CTQ = Childhood Trauma Questionnaire, SRSRQ = Stress-Reactive State Rumination Questionnaire, PANAS = Positive and Negative Affect Schedule. Please note that while subjective stress ratings were assessed every 15 min post TSST (0 min, 15 min, 30 min, 45 min, 60 min), salivary cortisol was only assessed at 0 min, 15 min, 30 min, 60 min. Tube icon: Flaticon.com.

*Procedures.* After inclusion in the study, participants completed a laboratory assessment where stress was induced using the TSST. Due to the demands of fNIRS, the TSST paradigm was partly adapted: First, participants rated their current stress levels using a Visual Analogue Scale (VAS) ranging from 0% to 100% which was repeatedly done during the experiment. All VAS-ratings were printed on one page so participants could consider their last ratings. Next, a first saliva sampling for cortisol assessment using Salivettes (Sarsted AG & Co, REF 51.1534.500) and blood sampling using 5.5 ml S-Monovettes (Sarsted AG & Co, REF 03.1628) was done. Then, another stress rating and several questionnaires were completed including demographic data, symptoms of depression using the Beck Depression Inventory (BDI-II; Hautzinger et al., 2009), social anxiety by means of the Liebowitz Social Anxiety Scale (Liebowitz, 1987), trait rumination using the Ruminative Response Scale (RRS; Nolen-Hoeksema and Morrow, 1991) and adverse childhood experiences using the Childhood Trauma Questionnaire (CTQ; Bernstein et al., 2003) while participants were prepared for the fNIRS and ECG assessment.^1^ Then, after another stress rating, a 7-min resting-state measurement was performed where participants were instructed to keep their eyes open and let their mind wander. Afterwards, stress and state ruminative processes during the last 7 min were measured using the stress-reactive state rumination questionnaire (SRSRQ). After collecting another salivary sample to assess cortisol, participants completed two control tasks preparing them for the arithmetic task of the TSST. For control task 1 (ctl1), participants read aloud number sequences from a page, for control task 2 (ctl2), they sequentially subtracted 13 from different starting points. Both tasks were completed in the presence of a friendly study nurse without time or social pressure. Similar number ranges compared to the TSST were used and each task consisted of 6 trials (lasting for 40 s, followed by a 20 s pause) in order to be able to average across trials in the analysis of the fNIRS data. After each task, a stress rating was given and after both, current mood was assessed using the Positive and Negative Affect Schedule (PANAS; Watson et al., 1988). Then, the stress induction followed, with two experimenters wearing white coats entering the room. Both remained socially non-responsive during the whole TSST. Participants were instructed to prepare for a speech about their personal strengths and qualifications in a mock job interview by taking notes. After 5 min, the experimenters took away the notes and asked the participant to begin while a video camera was switched on. After the 5-min speech, the arithmetic task of the TSST followed where participants calculated backwards in steps of 13 from different starting points (e.g. 1013) while holding eye-contact with one of the experimenters. In case an error was made, participants had to start all over again. After 6 min, the camera was switched off and the experimenters left the room while another stress rating, current mood (PANAS) and salivary cortisol sample were assessed. Following this, another resting-state measurement similar to the first one was conducted after which stress was assessed and the SRSRQ was completed. Furthermore, participants described their qualitative thought content during the second resting-state measurement using a custom open text questionnaire (see qualitative text analysis in methods section). Following this, after the end of the TSST and 15, 30 and 60 min following the TSST, salivary samples for cortisol assessment were collected. Additionally, another SRSRQ (post 45 min), and another PANAS (post 60 min) were assessed and another blood sample was collected (post 60 min) (see Fig. 1).

*fNIRS.* In order to assess oxygenated (HbO2) and deoxygenated hemoglobin (HbR) in the regions of interest (ROI): bilateral inferior frontal gyrus (IFG), DLPFC and somatosensory association cortex (SAC), we used the same fNIRS methodology as in our previous studies (Rosenbaum et al., 2018b, 2021). A detailed description can be found in the supplementary material.

*Salivary cortisol.* Using Salivettes (Sarsted AG & Co, REF 51.1534.500), we collected 6 salivary cortisol samples throughout the experiment. The samples were handled according to the manufacturer’s recommendations. After centrifuging for 2 min at 1000 g, an enzyme-linked immunosorbent assay (ELISA, IBL International, REF RE52611) was performed. As measurements took place during the whole day and cortisol is influenced by circadian rhythms, we regressed the effect of daytime out of the cortisol data and used the corrected data for our analysis.

*ECG.* Using two standard Ag/AgCl ring electrodes (8 mm diameter), we assessed a one-channel electrocardiogram. Electrodes were attached below the left costal arch, below the right collarbone and on the neck using Ten20 conductive paste after disinfecting the corresponding skin areas. With a sampling rate of 1000 Hz, heart rate was recorded using the Brain Products hard- and software (BrainAmp ExG amplifier and Brain Vision Recorder; Brain Products, Munich, Germany). Using BrainVision Analyzer 2.1 (Brain Products, Munich, Germany), preprocessing included bandpass filtering with a low cutoff at 1 Hz (time constant 0.03183099, 48 dB/oct) and a high cutoff at 30 Hz (48 dB/oct) (Kligfield et al., 2007). We applied a notch filter at 50 Hz due to power line artifacts. Then, for each condition separately, R-peaks were detected using the pulse artifact correction tool before we calculated the mean interval between subsequent R-peaks in beats per minute (bpm).

*Qualitative Text Analysis.* Following the second resting-state measurement and quantitative assessments, subjects completed an open text report regarding their experiences during the second resting-state measurement. Subjects were instructed to describe in detail what they had done and felt during the resting-state measurement. This qualitative data was analyzed by two independent raters (EJL and DR) that were blind towards diagnostic groups. A detailed description of the qualitative text analysis can be found in the supplemental material.

*Data Analysis.* Data analysis was done using SPSS (IBM Corp., Version 28.0). We separated the analysis into 3 sections: 1) Manipulation checks with respect to the stress induction to verify that the TSST procedure induced psychophysiological stress responses in the groups, 2) hypothesis-relevant tests and 3) the exploratory analysis with respect to the influence of clinical variables.

We analyzed performance during the TSST in terms of the calculations and errors during the arithmetic task averaged over the six blocks using repeated measures ANOVA with the between-subjects factor group (MDD vs HC) and the within-subjects factor condition (ctl1 vs. ctl2 vs. arithmetic task of the TSST). Behavioral data (subjective stress ratings, negative affect, and state rumination) was analyzed using repeated measures ANOVAs with the within-subjects factor time and between-subjects factor group (MDD vs. HC) as well as their interaction. Furthermore, we investigated Reliable Change Indexes for state rumination using the Reliable Change Index (RCI) (Jacobson and Truax, 1992) with the following formula: $RCI=\frac{y_{1,i}-y_{0,i}}{s_{Diff}}$ with $S_{Diff}=\sqrt{2*\left(S_{0}\sqrt{1-r_{yy}}\right)}²$ and $S_{0}$ as the Standard Deviation of the state rumination questionnaire pre TSST ($S_{0}=.94$) and $r_{yy}$ as the retest reliability ($r_{yy}=.92)$.

Ratings of the qualitative self-reports were analyzed with a MANOVA with the between-subjects factor group and the rated scales as dependent variables. Psychophysiological measures (salivary cortisol and heart rate) were analyzed using repeated measures ANOVAs with the factors group and time. Next, we analyzed relative changes in cortical oxygenation using repeated measures ANOVAs dependent on the ROI (lDLPFC, rDLPFC, lIFG, rIFG and SAC), condition (ctl1 vs. ctl2 vs. arithmetic task of the TSST) and group (MDD vs. HC) as well as their interactions.

As our main analysis showed an expected pattern of increased psychological and physiological stress parameters, but a to some extent additional unexpected pattern of depression-related effects (only main effect on state rumination) in comparison to our previous studies, we further performed an explorative analysis to check for influences through other clinical variables. Therefore, we conducted a hierarchical regression analysis in which state rumination reactivity scores (increases in state rumination) were predicted through (1) social anxiety, (2) number of depressive episodes, (3) the subscales of the childhood trauma scale (Häuser et al., 2011), (4) trait rumination, as assessed by the RRS, and (5) depression severity, as assessed by the BDI-II. Afterwards, we checked for differences in the fNIRS data in the significant predictors of the regression analysis, by using the significant predictors of the regression analysis as between-subject factors in a repeated measures ANOVA predicting fNIRS activation during the TSST (ctl1 vs. ctl2 vs. arithmetic task of the TSST) in the ROIs (lDLPFC, rDLPFC, lIFG, rIFG and SAC). Finally, we performed a subgroup analysis with small sample sizes to explore the effects of the significant variables. For this analysis we performed a repeated measures ANOVA with the within-subjects factor ROI and the between-subjects factor subgroup on reactivity measures (TSST-arithmetic task – ctl1) of fNIRS measured HbO2.

For all analyses we used polynomial contrasts as post-hoc contrasts. As heart rate is dependent on the posture of the participants (e.g. standing vs. sitting), we used t-tests as post-hoc tests and results were corrected for multiple comparisons with FDR correction (False-Discovery-Rate of 5%). Due to measurement artifacts in the ECG data, in 27 subjects out of 97 the data was excluded from the analysis. To check for influences of the exclusion of those subjects, we created a dataset with imputed data (SPSS analysis of missing values). All results were consistent between the data excluding subjects showing missing values and the fully imputed dataset. Therefore, in the following only the data of the original dataset (excluding subjects with missing data) is reported.

Note that finally, we reanalyzed our data using ANCOVA and MANCOVA with the covariates sex and age. The reported results in this paper remained stable when adding those variables as covariates.

## 3 Results

### 3.1 Manipulation checks

**Performance.** The analysis of calculation performance during the TSST showed a significant effect of condition (*F*(2,190) = 843.690, *p* < 0.001, $\eta_{p}^{2}$ = 0.90) and an interaction of group by condition (*F*(2,190) = 6.109, *p* < 0.01, $\eta_{p}^{2}$ = 0.06). Post-hoc tests showed that the group by condition interaction was characterized by different u-shaped changes in performance from ctl1 to the TSST for each group (*F*(1,95) = 7.905, *p* < 0.01, $\eta_{p}^{2}$ = 0.08). While both groups showed comparable performance during ctl1, HC performed better at ctl2 (on average 10.4 (*SD*_*HC*_ = 3.34) vs. 8.7 (*SD*_*MDD*_ = 3.6) calculations) and TSST (on average 12.5 (*SD*_*HC*_ = 4.3) vs. 10.9 (*SD*_*MDD*_ = 4.2)) calculations. With respect to errors, there were no differences between MDD and HC, however, the percentage of errors increased significantly from easy (ctl1) to more demanding conditions (ctl2 and TSST) (*F*(2,190) = 142.892, *p* < 0.001, $\eta_{p}^{2}$ = 0.60) in a linear manner (*F*(1,95) = 208.276, *p* < 0.001, $\eta_{p}^{2}$ = 0.68).

**Fig. 2:**
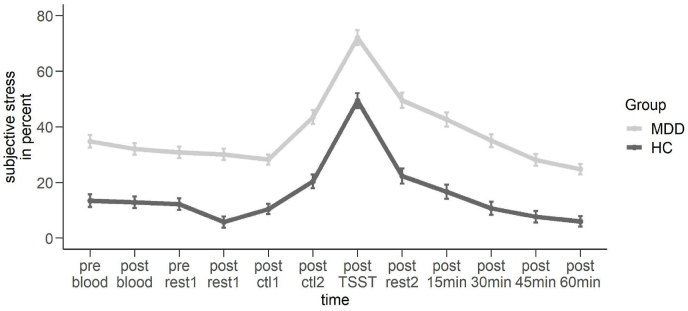
Subjective stress ratings in percent dependent on group. MDD = Major Depressive Disorder, HC = Healthy controls. Error bars show 1 standard error of the mean.

**Subjective stress.** By using a repeated measures ANOVA with the within-subjects factor time (VAS stress rating 1 to 12) and the between-subjects factor group (MDD vs. HC), we observed a significant main effect of time, *F*(4.76,447.60) = 75.505, *p* < 0.001, $\eta_{p}^{2}$ = 0.45, a significant main effect of group, *F*(1,94) = 70.588, *p* < 0.001, $\eta_{p}^{2}$ = 0.43, however no significant interaction of both factors (see Fig. 2). In general, MDD patients showed elevated levels of stress in comparison the HC and all subjects showed an expected increase in stress through the TSST.

**Negative affect.** A repeated measures ANOVA for negative mood (PANAS) dependent on time (post rest1, post TSST 0 min, post TSST 60 min) and group (MDD vs. HC) indicated a significant main effect of time, *F*(2,192) = 51.135, *p* < 0.001, $\eta_{p}^{2}$ = 0.35, and a significant main effect of group, *F*(1,96) = 69.589, *p* < 0.001, $\eta_{p}^{2}$ = 0.42, whereas their interaction did not yield significance. The main effect of group was characterized by an increased negative affect in the MDD group compared to HC. Considering within-subjects contrasts, time yielded a significant quadratic contrast, *F*(1,96) = 98.075, *p* < 0.001, $\eta_{p}^{2}$ = 0.51, indicating a significant increase in negative affect following the TSST and decrease 60 minutes after the TSST.

**Qualitative Reports.** Observer-rated qualitative reports were analyzed using a MANOVA with the between-subjects factor group and the rated scales as dependent variables. The analysis showed differences between the groups (*F*(15,80) = 3.587, *Λ* = 0.598, *p* < 0.001, $\eta_{p}^{2}$ = 0.40). Benjamini-Hochberg-corrected post-hoc univariate comparisons showed significantly higher levels in the MDD group of focus on negative affect (*F*(1,94) = 24.144, *p* < 0.001, $\eta_{p}^{2}$ = 0.20), ruminations on performance (*F*(1,94) = 11.698, *p* < 0.001, $\eta_{p}^{2}$ = 0.11), ruminations on consequences (*F*(1,94) = 11.400, *p* < 0.001, $\eta_{p}^{2}$ = 0.11), avoidance (*F*(1,94) = 7.943, *p* < 0.01, $\eta_{p}^{2}$ = 0.08) and suppression (*F*(1,94) = 6.675, *p* < 0.05, $\eta_{p}^{2}$ = 0.06), as well as lower levels of reframing (*F*(1,94) = 113.914, *p* < 0.001, $\eta_{p}^{2}$ = 0.13), distraction (*F*(1,94) = 8.345, *p* < 0.01, $\eta_{p}^{2}$ = 0.08) and mindfulness (*F*(1,94) = 4.447, *p* < 0.05, $\eta_{p}^{2}$ = 0.05).

**Fig. 3:**
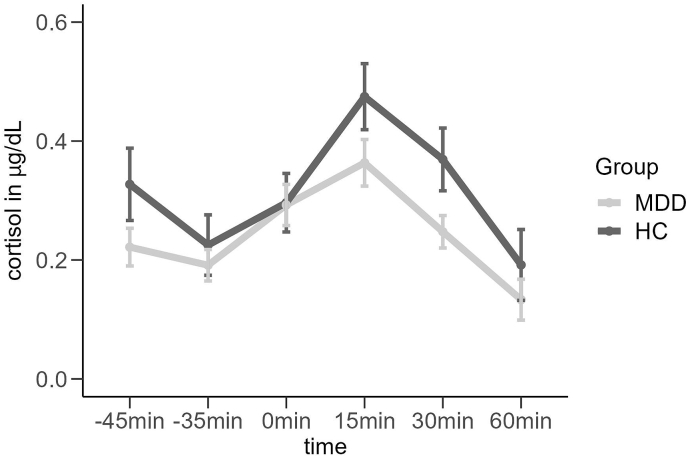
Mean cortisol levels dependent on group. MDD = Major Depressive Disorder, HC = Healthy controls. The x-axis represents the time relative to TSST completion: 0 min represents the end of the TSST, −45 min the first saliva collection. Error bars show 1 standard error of the mean.

**Cortisol.** Results of a repeated measurement ANOVA revealed a significant main effect of time (*F*(5,465) = 27.359, *p* < 0.001, $\eta_{p}^{2}$ = 0.23) and a group by time interaction (*F*(5,465) = 2.721, *p* < 0.05, $\eta_{p}^{2}$ = 0.03). Post-hoc analysis revealed a significantly different quadratic cortisol stress response between the MDD group and HC (*F*(1,93) = 6.152, *p* < 0.05, $\eta_{p}^{2}$ = 0.06) with MDD patients showing a blunted cortisol response following 15 min after the TSST (see Fig. 3).

**Fig. 4:**
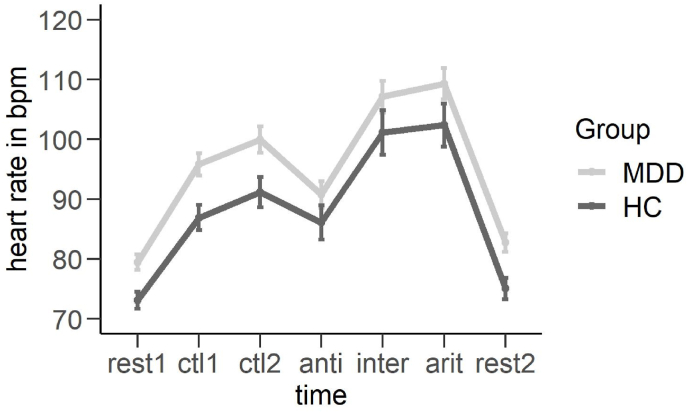
Mean heart rate in beats per minute (bpm) dependent on group. MDD = Major Depressive Disorder, HC = Healthy controls, rest1 = resting-state measurement 1, ctl1 = control task 1 (reading numbers), ctl2 = control task 2 (mental arithmetic without time or social stress), anti = anticipation phase of the job interview (preparation, taking notes), inter = job interview of the TSST, arit = arithmetic task of the TSST, rest2 = resting-state measurement 2. Error bars show 1 standard error of the mean.

**Heart rate.** Analyzing heart rate data, we observed a well-known effect of time (*F*(6,408) = 125.924, *p* < 0.001, $\eta_{p}^{2}$ = 0.64) and a main effect of group (*F*(1,68) = 6.037, *p* < 0.05, $\eta_{p}^{2}$ = 0.08). The main effect of group was characterized by a generally increased heart rate in the MDD group as compared to HC. Planned comparisons of the main effect of time revealed a consistent increase in heart rate during the TSST stress conditions (arithmetic task, interview task) as compared to the control conditions (ctl1, ctl2) (see Table 2 and Fig. 4).

**Table 2:** Planned comparisons of the main effect of time in case of the analysis of heart rate.

Comparison	*t*-value	*p*-value	Cohen's *d*
ctl1 vs. TSST-arith	*t*(80) = −9.092	*p* < 0.001	*d* = 1.01
ctl1 vs. TSST-interview	*t*(76) = −7.197	*p* < 0.001	*d* = 0.82
ctl2 vs. TSST-arith	*t*(77) = −7.360	*p* < 0.001	*d* = 0.83
ctl2 vs. TSST-interview	*t*(73) = −4.793	*p* < 0.001	*d* = 0.56

### 3.2 Hypothesis testing

**Fig. 5:**
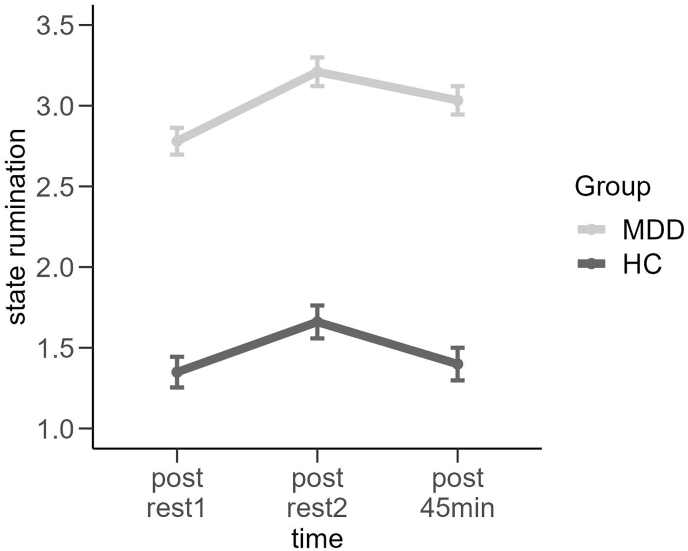
State rumination ratings dependent on group. MDD = Major Depressive Disorder, HC = Healthy controls. Error bars show 1 standard error of the mean.

**State rumination.** Next, we analyzed stress-induced ruminative processes as assessed using the SRSRQ. A repeated measures ANOVA dependent on time (post rest1 vs. post rest2 vs. post TSST 45 min) and group (HC vs. MDD) indicated significant main effects of time, *F*(1.76,167.03) = 17.575, *p* < 0.001, $\eta_{p}^{2}$ = 0.16 and group, *F*(1,95) = 193.463, *p* < 0.001, $\eta_{p}^{2}$ = 0.67, reflecting a general increase and higher levels of state rumination in the MDD group (see Fig. 5). The group by time interaction was not significant (F(2,190) = 1.329, p > 0.1, $\eta_{p}^{2}$ = 0.03). Post-hoc analysis revealed that both groups showed substantial increases in rumination after stress induction (MDD: *t*(54) = 3.886, *p* < 0.001, *d* = 0.54, HC: *t*(41) = 4.295, *p* < 0.001, *d* = 0.66).

However, the analysis of RCI showed that the MDD group had more reliable increases in state rumination than the HC (MDD = 29%, HC = 17%) from post rest1 to post rest2 through the TSST (χ^2^(1) = 2.745, *p*_onesided_<0.05), but at the same time also had more reliable decreases in state rumination than the HC (MDD = 10%, HC = 0%) (χ^2^(1) = 2.745, *p*_onesided_<0.05), (χ^2^(1) = 5.329, *p*_onesided_<0.05) (see Supplementary Table 5).

**Fig. 6:**
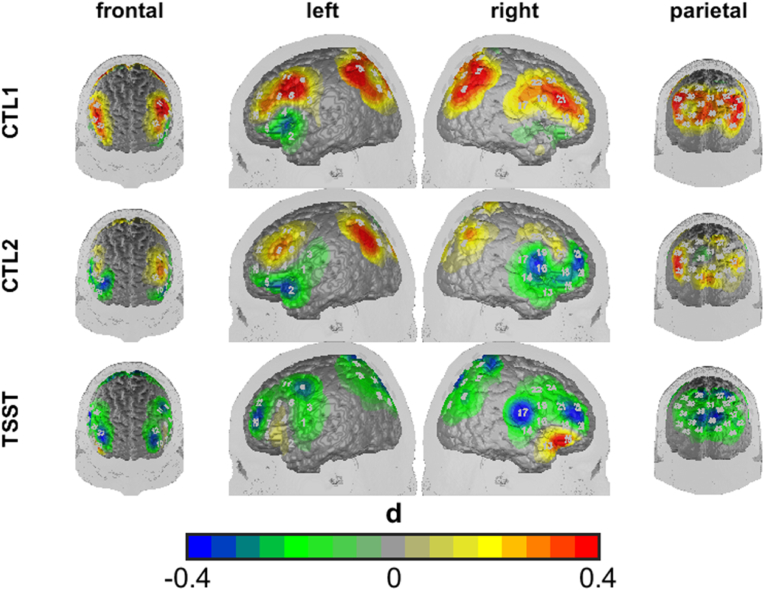
Differences between MDD and HC in mean cortical oxygenation during control task 1 (ctl1, reading numbers), control task 2 (ctl2, mental arithmetic without time or social stress) and the arithmetic task of the TSST (TSST, mental arithmetic under time or social stress). Cool colors indicate reduced HbO2 levels in the MDD group as compared to the HC group; warm colors vice versa. Differences are depicted in Cohen’s *d*. (For interpretation of the references to color in this figure legend, the reader is referred to the Web version of this article.)

**fNIRS.** Analyzing cortical oxygenation, we observed a significant main effect of condition (HbO2: *F*(2,190) = 4.760, *p* < 0.01, $\eta_{p}^{2}$ = 0.05; HbR: *F*(2,190) = 4.330, *p* < 0.05, $\eta_{p}^{2}$ = 0.04), ROI (HbO2: *F*(4,380) = 8.113, *p* < 0.001, $\eta_{p}^{2}$ = 0.08; HbR: *F*(4,380) = 2.066, *p* < 0.1, $\eta_{p}^{2}$ = 0.02) as well as a significant interaction of time and group, (HbO2: *F*(2,190) = 6.169, *p* < 0.01, $\eta_{p}^{2}$ = 0.06; HbR: *F*(2,190) = 1.175, *p* > 0.1, $\eta_{p}^{2}$ = 0.01). Post-hoc analysis showed a significant increase in HbO2 (and decrease in HbR) levels from control to stress conditions as indicated by linear contrasts (HbO2: *F*(1,95) = 7.758, *p* < 0.01, $\eta_{p}^{2}$ = 0.08; HbR: *F*(1,95) = 7.942, *p* < 0.01, $\eta_{p}^{2}$ = 0.08), as well as a significant linear contrast for the interaction effect of time and group, reflecting blunted HbO2 increases in the MDD group as compared to HC (HbO2: *F*(1,95) = 10.970, *p* < 0.001, $\eta_{p}^{2}$ = 0.10; HbR: *F*(1,95) = 0.162, *p* > 0.1, $\eta_{p}^{2}$ = 0.03) (see Fig. 6).

As both groups differed in their arithmetic performance, we further checked if the group effects on HbO2 levels stayed significant after controlling for arithmetic performance by adding arithmetic performance as a continuous covariate in a multilevel model. Results showed significant interactions of group*condition within the bilateral DLPFC, right IFG and SAC (see Supplementary Table 2).

**Exploratory analysis.** With respect to our exploratory analysis, in all regression models, social anxiety (*t* = 2.970, *p* < 0.01, *R*^*2*^ = 0.05) and self-reported emotional abuse (*t* = 2.546, *p* < 0.05, *R*^2^ = 0.05) during childhood were positively associated with stress-reactive state rumination, i.e. higher social anxiety and reported emotional abuse during childhood were significantly associated with higher increases in state rumination following the TSST.

Investigating the distribution of dichotomized relevant levels of childhood emotional abuse (CTQ emotional abuse >12) and social anxiety (LSAS >64) showed that nearly no subject in the HC group was socially anxious (*n* = 0) or had a history of emotional abuse (*n* = 3), but that in the MDD group *n* = 18 subjects (33.9 %) had a history of emotional abuse and *n* = 19 (35.8 %) were socially anxious (see Supplementary Table 4a-c). Moreover, within the MDD sample, social anxiety and emotional abuse were evenly distributed and not related to each other (*X*^*2*^(1) = 0.110, *p* > 0.1). Notably, with respect to reliable changes in state rumination through the TSST, the MDD group with emotional abuse was found to have more participants with reliable increases (50%) and fewer (i.e., none) with reliable decreases (0%) than the MDD group without emotional abuse (increases = 20%, decreases = 11%) (χ^2^(2) = 7.230), p > 0.05) (see Supplementary Table 6). In the following, we analyzed differences in TSST-related fNIRS activity using a repeated measurement ANOVA including three groups: HC, MDD-only, MDD-abuse/MDD-socially anxious.

Investigating the effects of emotional abuse on cortical brain oxygenation, we observed a two-way interaction of group by condition on HbO2 values (HbO2: F(4,178) = 4.237, *p* < 0.01, $\eta_{p}^{2}$ = 0.09; HbR: F(4,178) = 0.550, *p* > 0.1, $\eta_{p}^{2}$ = 0.01). FDR-corrected post-hoc analysis showed that the MDD-abuse group showed reduced cortical oxygenation during the TSST arithmetic task in comparison to the HC (HbO2: *t*(55) = 3.118, *p* < 0.001, *d* = 0.88; HbR: *t*(55) = 0.485, *p* > 0.1, *d* = 0.14) and MDD-no abuse group (HbO2: *t*(51) = 2.248, *p* < 0.05, *d* = 0.65; HbR: *t*(51) = 0.953, *p* > 0.1, *d* = 0.27). No differences were observed between the MDD-no abuse and HC group (HbO2: *t*(72) = 0.601, *p* > 0.1, *d* = 0.14; HbR: *t*(72) = 0.477, *p* > 0.1, *d* = 0.11).

Repeating the analysis with respect to social anxiety, we observed a significant group by condition interaction (HbO2: *F*(4,186) = 4.365, *p* < 0.01, $\eta_{p}^{2}$ = 0.09; HbR: *F*(4,178) = 2.757, *p* < 0.05, $\eta_{p}^{2}$ = 0.06). Post-hoc analysis showed significantly increased cortical oxygenation in the MDD-anxiety group during the TSST arithmetic task in comparison to the MDD-no anxiety group (HbO2: *t*(52) = 2.928, *p* < 0.01, *d* = 0.83; HbR: *t*(52) = 2.515, *p* < 0.05, *d* = 0.72) and non-significant differences compared to the HC (HbO2: *t*(59) = 0.607, *p* < 0.1, *d* = 0.17; HbR: *t*(59) = 1.375, *p* > 0.1, *d* = 0.38). Furthermore, HC showed higher cortical oxygenation than the MDD-no anxiety group (HbO2: *t*(75) = 3.337, *p* < 0.001, *d* = 0.76; HbR: *t*(75) = 1.064, *p* > 0.1, *d* = 0.24).

**Fig. 7:**
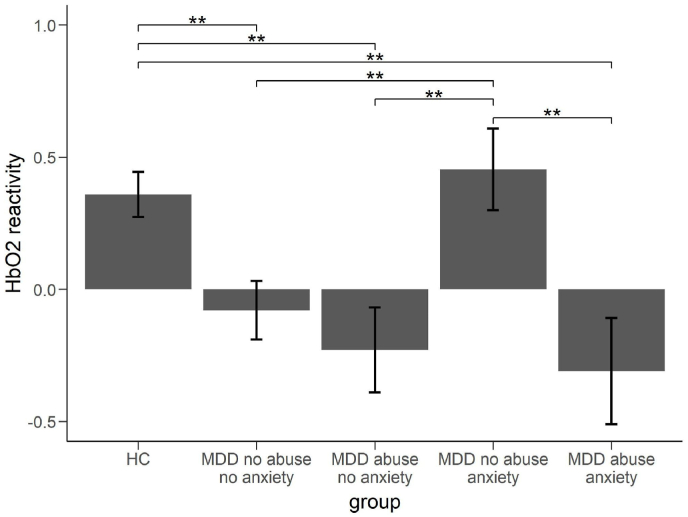
Differences in HbO2 reactivity (TSST arithmetic task vs. control task 1) between MDD with and without history of abuse and anxiety and HC. Shown is the mean CCN reactivity over all ROIs. All tests are FDR corrected. **p < 0.01. Error bars show 1 standard error of the mean.

To further investigate these differential effects of emotional abuse and social anxiety on brain activity in MDD, we explored small subgroups within the MDD patients: MDD-no abuse & no anxiety (*n* = 23), MDD abuse & no anxiety (*n* = 11), MDD no abuse & anxiety (*n* = 12), MDD abuse & anxiety (*n* = 7). A repeated measures ANOVA on the TSST-related increases in HbO2/decreases in HbR (TSST-arith - ctl1) in the different ROIs revealed a main effect of the factor group (HbO2: *F*(4,87) = 6.212, *p* < 0.001, $\eta_{p}^{2}$ = 0.22, HbR: *F*(4,87) = 2.440, *p* < 0.1, $\eta_{p}^{2}$ = 0.10). Post-hoc analysis showed significant differences between two homogenous groups: The groups of HC and MDD no abuse & anxiety showed significantly increased HbO2 reactivity scores compared to the other MDD groups: MDD no abuse & no anxiety, MDD abuse & no anxiety, MDD abuse & anxiety (see Fig. 7 and Supplementary Table 3).

## 4 Discussion

This study aimed to replicate our previous findings on stress-reactive rumination in MDD and further explore clinical characteristics (e.g. childhood trauma, social anxiety, number of depressive episodes) that might influence the expected prefrontal hypoactivation in the clinical sample. To this end, 55 patients with MDD and 42 HC were exposed to the TSST while cortical oxygenation was assessed using fNIRS. As expected, the TSST induced psychological as well as physiological stress. On a neural level, the arithmetic task of the TSST correlated with increased activity within the CCN. In line with our previous investigations (Rosenbaum et al., 2018b, 2021), MDD patients showed generally increased levels of subjective stress, negative mood and state rumination, but also worse performance during the TSST in terms of performed calculations. However, increases in state rumination were comparable between the MDD group and HC. This absent interaction was most probably due to the relatively large increase in state rumination in the HC as compared to our previous investigations (see Supplementary Fig. 1) (Rosenbaum et al., 2018b, 2021) and due to 10% (n = 4) of depressed subjects that showed a reliable decline in state rumination through the TSST. Indeed, when investigating RCI, the MDD group showed more reliable increases in state rumination than the HC. In fact, exclusion of the n = 4 depressed subjects that showed a decrease in state rumination would have resulted in a significant group interaction. On a physiological level, increased heart rates and blunted cortisol responses were observed in the clinical sample. In line with our hypothesis, MDD patients showed reduced cortical oxygenation during the TSST. However, this effect seemed to be in part independent from the state rumination increases as HC showed increased cortical oxygenation in prefrontal areas and increases in state rumination as well. Inferentially, the differences between the groups in cortical oxygenation were not associated with differences in stress-reactive rumination (as the groups did not differ in stress-reactive rumination), although the MDD group showed generally elevated state rumination levels and lower cortical oxygenation during stress. To further investigate factors that might contribute to the reduced stress-reactive prefrontal oxygenation in MDD and stress-reactive rumination, we performed an exploratory analysis. In this analysis, we first performed a regression analysis to investigate clinical factors that contribute to stress-reactive rumination. Secondly, we investigated the significant predictors with respect to their influence on cortical oxygenation during the TSST. This explorative analysis showed that social anxiety and self-reported retrospective emotional abuse were positively associated with increased stress-reactive rumination during the TSST. Especially the MDD subgroup with a history of emotional abuse showed an increased number of subjects (50%) with reliable increases in stress-reactive rumination. Furthermore, emotional abuse was associated with prefrontal hypoactivity during stress. In contrast, social anxiety was associated with increased prefrontal activity. Lastly, our final analysis showed that HC and MDD patients with social anxiety but no emotional abuse showed comparable increased CCN-activity during the TSST, whereas participants with only MDD and MDD patients with a history of emotional abuse - regardless of their anxiety level - showed a prefrontal hypoactivation during the TSST. Importantly, those results were based on a patient sample without diagnosis of Posttraumatic Stress Disorder (PTSD) or Social Anxiety Disorder. These differential activation patterns indicate an opposing effect of previous emotional abuse and anxiety on regulatory control exerted by the prefrontal cortex (PFC). This is in line with previous findings indicating - on the one hand - a disengagement of PFC-related regulatory responses in negative emotional situations in adolescents with a history of physical, sexual or emotional abuse (Wiegand et al., 2021) and - on the other hand - generally increased PFC activation in anxiety, based on recent meta-analytic data (Chavanne and Robinson, 2021) (for further discussion of these differing activation patterns also with respect to stress-reactive rumination, see below).

Taken together, in this study we could replicate our previous findings on the neural correlates of the TSST in HC and MDD patients. While in general the TSST is associated with increased oxygenation in the dorsal and ventral prefrontal cortex, in MDD and high trait ruminators those areas are hypoactivated (Henze et al., 2023; Rosenbaum et al., 2018, 2021). Further, in addition to physiological stress responses, the TSST induced subjective psychological stress-states, such as elevated negative affect and state rumination. Surprisingly, in this investigation we observed a comparatively high stress-reactive rumination response in the HC group. So far, it is not conclusive which variables might have contributed to this elevated stress-reactive rumination in the HC group. The observed effect was twice as high as in the HC in previous investigations (Rosenbaum et al., 2018b, 2021). Although speculative, first explanations could be attributed to (1) differences in the TSST interviewer constellation, (2) statistical chance and (3) general problems with psychological measures (e.g. biases, extreme answers, lack of interval scale). Although we performed the TSST set-up in this study as in our previous studies, the constellation of our TSST interviewers changed. In general, the TSST is a well-established study setup to reliably induce psychological and physiological stress on a population level. However, in our experience, there are of course differences in the “matching” of participants and TSST interviewers. In the present study, it might have happened that certain HC were matched towards a TSST interviewer group that triggered stress-reactive rumination in HC. Furthermore, varying effect sizes between studies might be a result of statistical chance. With respect to this point, one has to bear in mind that psychological state measures are mostly lacking very good reliability and precision, partly due to the nature of subjective ratings (e.g. tendencies to extreme answers, biases, dissimulation etc.) and lack of adequate interval scale criteria. In line with this, our analysis of observer-rated qualitative reports indeed showed differences between the groups in reported TSST-related ruminative content. Up to date, no appropriate measure of state-ruminative behavior exists, due to the nature of this *Qualia* phenomenon as rumination cannot be directly observed from an objective perspective, nor perfectly rated subjectively with regard to the strength of the subjective experience. It will be an important endeavor for future investigations to improve change-sensitive state-measurements of rumination to a point where very good reliability, precision and interval scale criteria are achieved.

Interestingly, our exploratory analysis showed that two factors - social anxiety and a self-reported history of emotional abuse - were positively associated with state rumination increases, but differentially associated with CCN activity. Contrary to studies in healthy subjects with childhood maltreatment (Zhong et al., 2020), a history of emotional abuse during childhood in MDD patients was associated with increased stress-reactive rumination and hypo-activity during stress in the PFC. Current scientific evidence shows that highly and especially chronic stressful experiences such as trauma in childhood and adolescence influence brain development (Lupien et al., 2009). Those developmental changes include structural changes such as reduced hippocampal volume (Andersen and Teicher, 2008) and brain volume (Bellis et al., 1999; Cohen et al., 2006; Morandotti et al., 2013) as well as changes in brain functioning such as hypoactivity during emotional processing (Fonzo et al., 2016; Harmelen et al., 2014) and resting-state functional connectivity (Sokołowski et al., 2022). Notably, a history of abuse during childhood is associated not only with depression but also other mental disorders such as PTSD (Zaidi and Foy, 1994), psychotic disorders (Chaiyachati and Gur, 2021), Borderline Personality Disorder (Ball and Links, 2009; Cattane et al., 2017) and other anxiety disorders (Cougle et al., 2010; Safren et al., 2002), and those disorders have been associated with prefrontal dysfunction as well (Husain et al., 2020, 2021; Koenen et al., 2001; Koike et al., 2022; Matsuo et al., 2003; Yokoyama et al., 2015). It will be an important scientific endeavor to investigate the associations of stress-reactive RNT, prefrontal hypoactivity and childhood maltreatment in populations with different mental disorders to investigate transdiagnostic neurophysiological phenotypes.

In line with our previous investigations, social anxiety was positively associated with increased state rumination (Laicher et al., 2022). However, in contrast to our previous findings, social anxiety was also positively associated with increased CCN-activity during stress. Noteworthy, in our previous investigation we re-analyzed data from *N* = 90 participants and social anxiety was more normally distributed, i.e. there were more subjects with low trait rumination scores but high social anxiety scores, while in the study at hand, high social anxiety was only found in patients with MDD. Therefore, the current results are only generalizable to socially anxious MDD patients. Since all participants were informed in advance that they would be exposed to a social stress situation, it is possible that the sample was further biased, as only those (highly socially anxious) patients who were confident of and committed to social exposure may have participated. The increased CCN-activity in this subgroup could therefore be interpreted as a correlate of compensation, or emotion regulation during exposure. In line with this interpretation, previous studies showed high CCN-activity during exposure therapy in specific phobia (Rosenbaum et al., 2020d) and during emotion regulation paradigms (Laicher et al., 2023; Rosenbaum et al., 2020b). Interestingly, the dissociation between social anxiety and childhood emotional abuse on prefrontal activity is well in line with a meta-analysis on brain functioning during emotional processing in PTSD, social anxiety and specific phobia: While all three disorders were associated with hyperactivity in the amygdala and insula, only PTSD was associated with hypo-activity in the anterior cingulate cortex and ventrolateral prefrontal cortex (Etkin and Wager, 2007). Correspondingly, we observed hypoactivation in MDD patients with abuse during childhood - a high risk factor for complex PTSD - during stress exposure but not in socially anxious MDD patients. Up to date, it remains unclear which features of PTSD lead to a hypoactivation of the prefrontal cortex and further research is needed to fill this gap. In future investigations it will be an interesting endeavor to explore whether hypoactivity in the prefrontal cortex is associated with certain clinical characteristics of psychopathology. For example, it might be possible that hypoactivity in the prefrontal cortex during stress is associated with dissociative symptoms or reduced controllability. Indeed, the current research suggests that certain subtypes of PTSD - i.e. PTSD with predominant hyperarousal and dissociative PTSD - have different neural correlates (Lanius et al., 2006). Furthermore, evidence from a treatment study showed that increased controllability of ruminative thoughts was associated with increased perfusion in the left DLPFC (Baeken et al., 2021). However, up to date the results of our exploratory analysis cannot be explained completely, and the above-given interpretations are to some extent speculative, which is why further research in this area is needed.

Interestingly, although different in their neural correlates, social anxiety and emotional abuse were both associated with increased stress-reactive rumination. From the data at hand, this dissociation cannot finally be explained. Based on our data, we would expect that both factors - emotional abuse and social anxiety - show a similar behavioral phenotype, while being associated with different effects on the neural level. Another, yet inclusive interpretation could be that both factors influence different factors of rumination. In a recent investigation on the factor structure of perseverative thought (Hallion et al., 2022), previously qualitatively described dimensions of rumination (Laicher et al., 2023; Rosenbaum et al., 2020a) were empirically confirmed. The five factor-model of perseverative thought identified five factors of perseverative thought: dyscontrol, self-focus, valence, interpersonal, and uncertainty. It is plausible that the identified subgroups of the study at hand might have different levels of stress-reactive rumination within those factors that “sum up” to equivalent levels of resulting rumination, e.g. social anxiety might be more related to the interpersonal factor and emotional abuse to dyscontrol.

Despite these promising findings some important limitations have to be kept in mind. First, as already noted, in this investigation social anxiety and emotional abuse were only observed within the clinical population. Further, these constructs were measured on a dimensional scale without a diagnosis of PTSD or social phobia. The resulting subgroups of patients in the last exploratory analysis consisted of few subjects, further limiting the stability and generalizability of the results. In the same context, we propose the development of state rumination measures that include all five factors of perseverative thought. The currently used questionnaire in this investigation focuses predominantly on facets of uncontrollability, self-focus and valence and might therefore be limited in the investigation of ruminative-perseverative processes in social anxiety. With respect to neurophysiology, the well-known drawbacks of fNIRS must also be kept in mind, as the penetration depth is limited to cortical areas (Haeussinger et al., 2011). Therefore, subcortical areas of emotional processing such as the limbic system could not be investigated. Finally, for different reasons (different answering times between MDD and HC, CORONA pandemic regulations, different number of assessments in the lab), the MDD group did answer some baseline questionnaires at home, while the HC completed them in the laboratory. Despite this difference in the experimental procedure, baseline levels in the dependent variables seemed to be unaffected when compared to a previous study of our group (see Supplementary Fig. 1).

Nonetheless, the results of this study are supported by other investigations using different paradigms of emotional processing and give future directions for specific clinical populations with respect to stress-reactive rumination. To the knowledge of the authors, this is the first study that investigates the influence of emotional abuse and social anxiety in MDD on state rumination and CCN activity during an experimental stress paradigm. In conclusion, we were able to replicate our previous findings with respect to stress-reactive prefrontal hypoactivity in MDD and stress-reactive rumination. Our exploratory analysis further suggests different phenotypes of stress-reactive hypoactivity in MDD dependent on a history of childhood emotional abuse and social anxiety levels. In future studies those predictors should be investigated in different mental disorders to check for a common transdiagnostic phenotype that could be targeted through neurophysiological (e.g. transcranial theta-burst stimulation) and psychotherapeutic (e.g. emotion regulation training) interventions.

## Significance statement

The results of our study replicate prior research, showing hypoactivity in the Cognitive Control Network as well as increased levels of state rumination in individuals with Major Depressive Disorder during acute stress in comparison to healthy controls. Our study contributes to the existing literature by investigating MDD-specific variables that may explain the neurophysiological effects. The results of this study suggest a strong association between traumatic experiences during childhood and prefrontal hypoactivity in MDD, emphasizing the existence of different neurophysiological phenotypes of certain MDD subgroups. These results inform the practitioner on the nature of depressive rumination as well as potential targets of psychotherapeutic treatment and neuro-modulation.

## Financial disclosures

This research was partly supported by fortüne research grant no 2570-1-0. No author reported conflicts of interest.

## CRediT authorship contribution statement

**David Rosenbaum:** Writing – original draft, Supervision, Software, Project administration, Methodology, Funding acquisition, Formal analysis, Conceptualization. **Isabell Int-Veen:** Writing – original draft, Visualization, Investigation. **Hendrik Laicher:** Writing – review & editing, Project administration, Investigation. **Leonie Woloszyn:** Writing – review & editing, Investigation. **Ariane Wiegand:** Writing – review & editing, Formal analysis. **Sandra Ladegast:** Writing – review & editing, Investigation. **Ute Eßer:** Writing – review & editing, Investigation. **Agnes Kroczek:** Writing – review & editing, Investigation. **Daniel Sippel:** Writing – review & editing, Investigation. **Sebastian Menkor:** Writing – review & editing, Investigation. **Glenn Lawyer:** Writing – review & editing, Software. **Francesco Albasini:** Writing – review & editing, Investigation. **Christian Frischholz:** Writing – review & editing, Investigation. **Rainald Mössner:** Writing – review & editing, Resources. **Vanessa Nieratschker:** Writing – review & editing, Resources. **Elisabeth J. Leehr:** Writing – review & editing, Formal analysis. **Julian Rubel:** Writing – review & editing, Formal analysis. **Andreas J. Fallgatter:** Writing – review & editing, Supervision. **Ann-Christine Ehlis:** Writing – original draft, Validation, Supervision, Resources, Project administration, Methodology, Funding acquisition, Formal analysis, Conceptualization.

## Declaration of competing interest

The authors declare that they have no known competing financial interests or personal relationships that could have appeared to influence the work reported in this paper.

## Data Availability

Data will be made available on request.

## Footnotes

1 Please note that MDD patients completed the questionnaire assessing demographic data, RRS and CTQ at home while only the BDI-II was assessed at the laboratory for the sake of time efficiency.

## References

[bib1] Andersen S.L., Teicher M.H. (2008). Stress, sensitive periods and maturational events in adolescent depression. Trends Neurosci..

[bib2] Baeken C., Wu G.-R., Rogiers R., Remue J., Lemmens G.M., Raedt R.D. (2021). Cognitive behavioral based group psychotherapy focusing on repetitive negative thinking: decreased uncontrollability of rumination is related to brain perfusion increases in the left dorsolateral prefrontal cortex. J. Psychiatr. Res..

[bib3] Ball J.S., Links P.S. (2009). Borderline personality disorder and childhood trauma: evidence for a causal relationship. Curr. Psychiatr. Rep..

[bib4] Ball T.M., Ramsawh H.J., Campbell-Sills L., Paulus M.P., Stein M.B. (2013). Prefrontal dysfunction during emotion regulation in generalized anxiety and panic disorders. Psychol. Med..

[bib5] Bean C.A.L., Heggeness L.F., Kalmbach D.A., Ciesla J.A. (2020). Ruminative inertia and its association with current severity and lifetime course of depression. Clin. Psychol. Sci..

[bib6] Bellis M.D., Baum A.S., Birmaher B., Keshavan M., Eccard C.H., Boring A.M., Jenkins F.J., Ryan N.D. (1999). AE bennett research award. Dev. Traumatol. Part Biol. Stress Syst. Biol. Psychaitr..

[bib7] Bernstein D.P., Stein J.A., Newcomb M.D., Walker E., Pogge D., Ahluvalia T., Stokes J., Handelsman L., Medrano M., Desmond D., Zule W. (2003). Development and validation of a brief screening version of the Childhood Trauma Questionnaire. Child Abuse Negl..

[bib8] Cattane N., Rossi R., Lanfredi M., Cattaneo A. (2017). Borderline personality disorder and childhood trauma: exploring the affected biological systems and mechanisms. BMC Psychiatr..

[bib9] Chaiyachati B.H., Gur R.E. (2021). Effect of child abuse and neglect on schizophrenia and other psychotic disorders. Pharmacol. Biochem. Behav..

[bib10] Chavanne A.V., Robinson O.J. (2021). The overlapping neurobiology of induced and pathological anxiety: a meta-analysis of functional neural activation. Am. J. Psychiatr..

[bib11] Cohen R.A., Grieve S., Hoth K.F., Paul R.H., Sweet L., Tate D., Gunstad J., Stroud L., McCaffery J., Hitsman B., Niaura R., Clark C.R., MacFarlane A., Bryant R., Gordon E., Williams L.M. (2006). Early life stress and morphometry of the adult anterior cingulate cortex and caudate nuclei. Biol. Psychiatr..

[bib12] Connolly S.L., Alloy L.B. (2017). Rumination interacts with life stress to predict depressive symptoms: an ecological momentary assessment study. Behav. Res. Ther..

[bib13] Cougle J.R., Timpano K.R., Sachs-Ericsson N., Keough M.E., Riccardi C.J. (2010). Examining the unique relationships between anxiety disorders and childhood physical and sexual abuse in the National Comorbidity Survey-Replication. Psychiatr. Res..

[bib14] De Wandel L., De Smet S., Pulopulos M.M., Lemmens G.M.D., Hidalgo V., Salvador A., Vanderhasselt M.-A., Pruessner J., Baeken C. (2023). The effects of left dorsolateral prefrontal transcranial direct current stimulation on episodic future thinking following acute psychosocial stress. Memory.

[bib15] Dedovic Katarina, Renwick Robert, Mahani Najmeh Khalili, Engert Veronika, Lupien Sonia J., Pruessner Jens C. (2005). The Montreal Imaging Stress Task: using functional imaging to investigate the effects of perceiving and processing psychosocial stress in the human brain. J. Psychiatry Neurosci..

[bib16] DeRosse P., Nitzburg G.C., Kompancaril B., Malhotra A.K. (2014). The relation between childhood maltreatment and psychosis in patients with schizophrenia and non-psychiatric controls. Schizophr. Res..

[bib17] Ehring T., Watkins E.R. (2008). Repetitive negative thinking as a transdiagnostic process. Int. J. Cognit. Ther..

[bib18] Etkin A., Wager T.D. (2007). Functional neuroimaging of anxiety: a meta-analysis of emotional processing in PTSD, social anxiety disorder, and specific phobia. Am. J. Psychiatr..

[bib19] First M., Williams J., Karg R., Spitzer R. (2015).

[bib20] Fonzo G.A., Ramsawh H.J., Flagan T.M., Simmons A.N., Sullivan S.G., Allard C.B., Paulus M.P., Stein M.B. (2016). Early life stress and the anxious brain: evidence for a neural mechanism linking childhood emotional maltreatment to anxiety in adulthood. Psychol. Med..

[bib21] Fowler C.H., Miernicki M.E., Rudolph K.D., Telzer E.H. (2017). Disrupted amygdala-prefrontal connectivity during emotion regulation links stress-reactive rumination and adolescent depressive symptoms. Dev. Cogn. Neurosci..

[bib22] Gardner M.J., Thomas H.J., Erskine H.E. (2019). The association between five forms of child maltreatment and depressive and anxiety disorders: a systematic review and meta-analysis. Child Abuse Negl..

[bib23] Gibb B.E., Alloy L.B., Abramson L.Y., Rose D.T., Whitehouse W.G., Donovan P., Hogan M.E., Cronholm J., Tierney S. (2001). History of childhood maltreatment, negative cognitive styles, and episodes of depression in adulthood. Cognit. Ther. Res..

[bib24] Gibb B., Alloy L., Abramson L., Marx B. (2003). Childhood maltreatment and maltreatment‐specific inferences: a test of Rose and Abramson’s (1992) extension of the hopelessness theory. Cognit. Emot..

[bib25] Grassi-Oliveira R., Stein L.M. (2008). Childhood maltreatment associated with PTSD and emotional distress in low-income adults: the burden of neglect. Child Abuse Negl..

[bib26] Groenewold N.A., Opmeer E.M., De Jonge P., Aleman A., Costafreda S.G. (2013). Emotional valence modulates brain functional abnormalities in depression: evidence from a meta-analysis of fMRI studies. Neurosci. Biobehav. Rev..

[bib27] Haeussinger F.B., Heinzel S., Hahn T., Schecklmann M., Ehlis A.-C., Fallgatter A.J. (2011). Simulation of near-infrared light absorption considering individual head and prefrontal cortex anatomy: implications for optical neuroimaging. PLoS One.

[bib28] Hallion L.S., Wright A.G.C., Joormann J., Kusmierski S.N., Coutanche M.N., Caulfield M.K. (2022). A five-factor model of perseverative thought. J. Psychopathol. Clin. Sci..

[bib29] Harmelen A.-L., Tol M.-J., Dalgleish T., Wee N.J.A., Veltman D.J., Aleman A., Spinhoven P., Penninx B.W.J.H., Elzinga B.M. (2014). Hypoactive medial prefrontal cortex functioning in adults reporting childhood emotional maltreatment. Soc. Cognit. Affect Neurosci..

[bib30] Häuser W., Schmutzer G., Brähler E., Glaesmer H. (2011). Misshandlungen in Kindheit und Jugend: Ergebnisse einer Umfrage in einer repräsentativen Stichprobe der deutschen Bevölkerung. Dtsch Arztebl Int.

[bib31] Hautzinger M., Keller F., Kühner C. (2009).

[bib33] Henze G.-I., Konzok J., Kreuzpointner L., Bärtl C., Peter H., Giglberger M., Streit F., Kudielka B.M., Kirsch P., Wüst S. (2020). Increasing deactivation of limbic structures over psychosocial stress exposure time. Biol. Psychiatry Cogn. Neurosci. Neuroimaging.

[bib34] Henze G.-I., Rosenbaum D., Bärtl C., Laicher H., Konzok J., Kudielka B.M., Fallgatter A.J., Wüst S., Ehlis A.-C., Kreuzpointner L. (2023). Comparing two psychosocial stress paradigms for imaging environments – ScanSTRESS and fNIRS-TSST: correlation structures between stress responses. Behav. Brain Res..

[bib35] Husain S.F., Tang T.-B., Yu R., Tam W.W., Tran B., Quek T.T., Hwang S.-H., Chang C.W., Ho C.S., Ho R.C. (2020). Cortical haemodynamic response measured by functional near infrared spectroscopy during a verbal fluency task in patients with major depression and borderline personality disorder. EBioMedicine.

[bib36] Husain S.F., Tang T.-B., Tam W.W., Tran B.X., Ho C.S., Ho R.C. (2021). Cortical haemodynamic response during the verbal fluency task in patients with bipolar disorder and borderline personality disorder: a preliminary functional near-infrared spectroscopy study. BMC Psychiatr..

[bib37] Int-Veen I., Fallgatter A.J., Ehlis A.-C., Rosenbaum D. (2023). Prefrontal hypoactivation induced via social stress is more strongly associated with state rumination than depressive symptomatology. Sci. Rep..

[bib38] Jacobs R.H., Jenkins L.M., Gabriel L.B., Barba A., Ryan K.A., Weisenbach S.L., Verges A., Baker A.M., Peters A.T., Crane N.A., Gotlib I.H., Zubieta J.-K., Phan K.L., Langenecker S.A., Welsh R.C. (2014). Increased coupling of intrinsic networks in remitted depressed youth predicts rumination and cognitive control. PLoS One.

[bib39] Jacobson N.S., Truax P. (1992). Methodological Issues & Strategies in Clinical Research.

[bib40] Kenwood M.M., Kalin N.H., Barbas H. (2022). The prefrontal cortex, pathological anxiety, and anxiety disorders. Neuropsychopharmacology.

[bib41] Kirschbaum C., Pirke K.M., Hellhammer D. (1993). The ‘trier social stress test’ – a tool for investigating psychobiological stress responses in a laboratory setting. Neuropsychobiology.

[bib42] Kligfield P., Gettes L.S., Bailey J.J., Childers R., Deal B.J., Hancock E.W., van H.G., Kors J.A., Macfarlane P., Mirvis D.M., Pahlm O., Rautaharju P., Wagner G.S. (2007). Recommendations for the standardization and interpretation of the electrocardiogram. J. Am. Coll. Cardiol..

[bib43] Koenen K.C., Driver K.L., Oscar-Berman M., Wolfe J., Folsom S., Huang M.T., Schlesinger L. (2001). Measures of prefrontal system dysfunction in posttraumatic stress disorder. Brain Cognit..

[bib44] Koike S., Sakakibara E., Satomura Y., Sakurada H., Yamagishi M., Matsuoka J., Okada N., Kasai K. (2022). Shared functional impairment in the prefrontal cortex affects symptom severity across psychiatric disorders. Psychol. Med..

[bib45] Laicher H., Int-Veen I., Torka F., Kroczek A., Bihlmaier I., Storchak H., Velten-Schurian K., Dresler T., Täglich R., Fallgatter A.J., Ehlis A.-C., Rosenbaum D. (2022). Trait rumination and social anxiety separately influence stress-induced rumination and hemodynamic responses. Sci. Rep..

[bib46] Laicher H., Int-Veen I., Woloszyn L., Wiegand A., Kroczek A., Sippel D., Leehr E.J., Lawyer G., Albasini F., Frischholz C., Mössner R., Nieratschker V., Rubel J., Fallgatter A., Ehlis A.-C., Rosenbaum D. (2023). In situ fNIRS measurements during cognitive behavioral emotion regulation training in rumination-focused therapy: a randomized-controlled trial. NeuroImage Clin..

[bib47] Lanius R.A., Bluhm R., Lanius U., Pain C. (2006). A review of neuroimaging studies in PTSD: heterogeneity of response to symptom provocation. J. Psychiatr. Res..

[bib48] Liebowitz M.R. (1987). Liebowitz Social Anxiety Scale (LSAS) [Database record]. APA PsycTests. doi:10.1037/t07671-000.

[bib49] Lupien S.J., McEwen B.S., Gunnar M.R., Heim C. (2009). Effects of stress throughout the lifespan on the brain, behaviour and cognition. Nat. Rev. Neurosci..

[bib50] Matsuo K., Taneichi K., Matsumoto A., Ohtani T., Yamasue H., Sakano Y., Sasaki T., Sadamatsu M., Kasai K., Iwanami A., Asukai N., Kato N., Kato T. (2003). Hypoactivation of the prefrontal cortex during verbal fluency test in PTSD: a near-infrared spectroscopy study. Psychiatry Res. Neuroimaging..

[bib51] Ming Q., Zhong X., Zhang X., Pu W., Dong D., Jiang Y., Gao Y., Wang X., Detre J.A., Yao S., Rao H. (2017). State-independent and dependent neural responses to psychosocial stress in current and remitted depression. Am. J. Psychiatr..

[bib52] Morandotti N., Dima D., Jogia J., Frangou S., Sala M., Vidovich G.Z.D., Lazzaretti M., Gambini F., Marraffini E., d’Allio G., Barale F., Zappoli F., Caverzasi E., Brambilla P. (2013). Childhood abuse is associated with structural impairment in the ventrolateral prefrontal cortex and aggressiveness in patients with borderline personality disorder. Psychiatry Res. Neuroimaging..

[bib53] Nanni V., Uher R., Danese A. (2012). Childhood maltreatment predicts unfavorable course of illness and treatment outcome in depression: a meta-analysis. Am. J. Psychiatr..

[bib54] Nolen-Hoeksema S., Morrow J. (1991). A prospective study of depression and posttraumatic stress symptoms after a natural disaster: the 1989 Loma Prieta earthquake. J. Pers. Soc. Psychol..

[bib55] Pulopulos M.M., Hoorelbeke K., Vandenbroucke S., Durme K., Hooley J.M., Raedt R. (2022). The interplay between self-esteem, expectancy, cognitive control, rumination, and the experience of stress: a network analysis. Curr. Psychol..

[bib56] Rosenbaum David, Maier M.J., Hudak J., Metzger F.G., Wells A., Fallgatter A.J., Ehlis A.-C. (2018). Neurophysiological correlates of the attention training technique: a component study. NeuroImage Clin..

[bib57] Rosenbaum D., Hilsendegen P., Thomas M., Haeussinger F.B., Metzger F.G., Nuerk H.-C., Fallgatter A.J., Nieratschker V., Ehlis A.-C. (2018). Cortical hemodynamic changes during the trier social stress test: an fNIRS study. Neuroimage.

[bib58] Rosenbaum D., Thomas M., Hilsendegen P., Metzger F.G., Haeussinger F.B., Nuerk H.-C., Fallgatter A.J., Nieratschker V., Ehlis A.-C. (2018). Stress-related dysfunction of the right inferior frontal cortex in high ruminators: an fNIRS study. NeuroImage Clin..

[bib59] Rosenbaum D., Int-Veen I., Kroczek A., Hilsendegen P., Velten-Schurian K., Bihlmaier I., Fallgatter A.J., Ehlis A.-C. (2020). Amplitude of low frequency fluctuations (ALFF) of spontaneous and induced rumination in major depression: an fNIRS study. Sci. Rep..

[bib60] Rosenbaum D., Kroczek A.M., Hudak J., Rubel J., Maier M.J., Sorg T., Weisbender L., Goldau L., Mennin D., Fresco D.M., Fallgatter A.J., Ehlis A.-C. (2020). Neural correlates of mindful emotion regulation in high and low ruminators. Sci. Rep..

[bib61] Rosenbaum D., Leehr E.J., Kroczek A., Rubel J.A., Int-Veen I., Deutsch K., Maier M.J., Hudak J., Fallgatter A.J., Ehlis A.-C. (2020). Neuronal correlates of spider phobia in a combined fNIRS-EEG study. Sci. Rep..

[bib62] Rosenbaum D., Leehr E.J., Rubel J., Maier M.J., Pagliaro V., Deutsch K., Hudak J., Metzger F.G., Fallgatter A.J., Ehlis A.-C. (2020). Cortical oxygenation during exposure therapy – in situ fNIRS measurements in arachnophobia. NeuroImage Clin..

[bib63] Rosenbaum D., Int-Veen I., Laicher H., Torka F., Kroczek A., Rubel J., Lawyer G., Bürger Z., Bihlmaier I., Storchak H., Velten-Schurian K., Dresler T., Täglich R., Schopp B., Nürk H.-C., Derntl B., Nieratschker V., Fallgatter A.J., Ehlis A.-C. (2021). Insights from a laboratory and naturalistic investigation on stress, rumination and frontal brain functioning in MDD: an fNIRS study. Neurobiol. Stress.

[bib64] Rosenbaum D., Int-Veen I., Rubel J., Laicher H., Kroczek A., Lawyer G., Fallgatter A.J., Ehlis A.-C. (2022). Associations of different emotion regulation strategies with coping-efficacy, rumination and stress. Cognit. Ther. Res..

[bib65] Ruscio A.M., Gentes E.L., Jones J.D., Hallion L.S., Coleman E.S., Swendsen J. (2015). Rumination predicts heightened responding to stressful life events in major depressive disorder and generalized anxiety disorder. J. Abnorm. Psychol..

[bib66] Safren S.A., Gershuny B.S., Marzol P., Otto M.W., Pollack M.H. (2002). History of childhood abuse in panic disorder, social phobia, and generalized anxiety disorder. J. Nerv. Ment. Dis..

[bib67] Siegle G.J., Steinhauer S.R., Thase M.E., Stenger V.A., Carter C.S. (2002). Can’t shake that feeling: event-related fMRI assessment of sustained amygdala activity in response to emotional information in depressed individuals. Biol. Psychiatr..

[bib68] Smith J.M., Alloy L.B. (2009). A roadmap to rumination: a review of the definition, assessment, and conceptualization of this multifaceted construct. Clin. Psychol. Rev..

[bib69] Sokołowski A., Kowalski J., Dragan M. (2022). Neural functional connectivity during rumination in individuals with adverse childhood experiences. Eur. J. Psychotraumatol..

[bib70] Speicher N., Henze G.-I., Bärtl C., Böhm F., Sommer M., Wüst S., Kudielka B.M. (2023). Neural correlates of everyday moral decision-making: an exploratory ScanSTRESS study. Psychol. Neurosci..

[bib71] Thai M., Schreiner M.W., Mueller B.A., Cullen K.R., Klimes-Dougan B. (2021). Coordination between frontolimbic resting state connectivity and hypothalamic–pituitary–adrenal axis functioning in adolescents with and without depression. Psychoneuroendocrinology.

[bib72] Watson D., Clark L.A., Tellegen A. (1988). Development and validation of brief measures of positive and negative affect: the PANAS scales. J. Pers. Soc. Psychol..

[bib73] Weber D.A., Reynolds C.R. (2004). Clinical perspectives on neurobiological effects of psychological trauma. Neuropsychol. Rev..

[bib74] Wiegand A., Munk M.H.J., Drohm S., Fallgatter A.J., MacIsaac J.L., Kobor M.S., Nieratschker V., Kreifelts B. (2021). Neural correlates of attentional control in social anxiety disorder: the impact of early-life adversity and DNA methylation. J. Psychiatry Neurosci..

[bib75] Witt S.T., Van Ettinger-Veenstra H., Salo T., Riedel M.C., Laird A.R. (2021). What executive function network is that? An image-based meta-analysis of network labels. Brain Topogr..

[bib76] Witte S., Baeken C., Pulopulos M.M., Josephy H., Schiettecatte J., Anckaert E., Raedt R., Vanderhasselt M.-A. (2020). The effect of neurostimulation applied to the left dorsolateral prefrontal cortex on post-stress adaptation as a function of depressive brooding. Prog. Neuro-Psychopharmacol. Biol. Psychiatry.

[bib77] Yokoyama C., Kaiya H., Kumano H., Kinou M., Umekage T., Yasuda S., Takei K., Nishikawa M., Sasaki T., Nishimura Y., Hara N., Inoue K., Kaneko Y., Suzuki S., Tanii H., Okada M., Okazaki Y. (2015). Dysfunction of ventrolateral prefrontal cortex underlying social anxiety disorder: a multi-channel NIRS study. NeuroImage Clin..

[bib78] Zaidi L.Y., Foy D.W. (1994). Childhood abuse experiences and combat-related PTSD. J. Trauma Stress.

[bib79] Zhong X., Ming Q., Dong D., Sun X., Cheng C., Xiong G., Li C., Zhang X., Yao S. (2020). Childhood maltreatment experience influences neural response to psychosocial stress in adults: an fMRI study. Front. Psychol..

