# Supplementary material for: Neural correlates of stress-reactive rumination in depression – The role of childhood trauma and social anxiety
Source: Neurobiol Stress. 2024 May 9;31:100640. doi: 10.1016/j.ynstr.2024.100640 (PMC11127161; doi:10.1016/j.ynstr.2024.100640)
Supplement: Multimedia component 1 [file mmc1.docx]

**Supplemental Material**

**fNIRS**: We used a continuous-wave multichannel NIRS system (ETG-4000 Optical Topography System; Hitachi Medical Co., Japan) with a temporal resolution of 10 Hz and a semiconductor laser and avalanche diodes (wavelengths: 695±20 and 830±20 nm). The measurement was conducted using the probeset placement utilized in our previous studies, monitoring cortical activity through two frontal probesets with 9 optodes each, as well as one probeset with 15 optodes covering the parietal ROI. These regions are as follows: Bilateral inferior frontal gyrus (IFG), bilateral dorsolateral prefrontal cortex (DLPFC) and somatosensory association cortex (SAC). Accordingly, the assessment utilized a total of 33 optodes, of which 18 were emitting and 15 receiving. The optodes were positioned on EEG caps (easycap) with 3 cm gaps and additionally stabilized by sponge rings. The exact optode position was adjusted according to electrode positions Fpz and Cz conforming to the 10-20-system [(Jasper, 1958)](https://www.zotero.org/google-docs/?mJwNp3). fNIRS data was pre-processed using MATLAB 2017a [(MathWorks Inc, 2017)](https://www.zotero.org/google-docs/?pSvcni). First, channels that indicated artifacts in the fNIRS signal were interpolated and a bandpass filter (0.01-0.1 Hz) was applied for both oxygenated and deoxygenated signals. Furthermore, data was corrected for high amplitude movement artifacts through Temporal Derivative Distribution Repair (TDDR) correction (Fishburn et al., 2019). In order to improve the signal further, a correlation-based signal improvement (CBSI) was conducted (Cui et al., 2010). A second interpolation process was then performed for signal artifacts resulting from these previous analysis steps for the CBSI-corrected oxygenated signal only. A last interpolation procedure was based on visual inspection of single artifact-laden channels. The global signal was reduced through application of a PCA-based Gaussian kernel filter (Zhang et al., 2016). Lastly, we calculated event-related averages separately for each region of interest dependent on the experimental task (ctl1, ctl2 and arithmetic task of the TSST) by calculating the mean over the six 40 s trials, respectively using a 5 s baseline correction. In the analysis we primarily report the results of the CBSI corrected oxygenated blood level (HbO2), however statistical parameters for the deoxygenated blood levels (HbR) are also reported.

***CTQ.*** For assessing adverse childhood experiences at the age of 0–17 years, we used the 28-item self-report Childhood Trauma Questionnaire (CTQ [Bernstein et al., 2003)](https://www.zotero.org/google-docs/?chKbuo). The CTQ comprises 25 clinical items which are part of 5 subscales (emotional abuse, physical abuse, sexual abuse, emotional neglect, and physical neglect) and three validity items to screen denial which are rated on 5-point Likert Scales ranging from 1 = "never true'' to 5 = "very often true'' and resulting in a total score ranging from 25–125. Using structured interviews in adolescent psychiatric patients (*N* = 179) applied by psychotherapists and data of child protective investigations, good criterion-related validity has been observed (Bernstein et al., 2003). In clinical and community samples, comparable internal consistencies of the subscales have been observed ranging from $\alpha$ = .61 (physical neglect) to $\alpha$ = .95 (sexual abuse). These findings could be replicated by other researchers (Bader et al., 2009; Burns et al., 2012, 2010; Huh et al., 2017; Klinitzke et al., 2012; Scher et al., 2001; Wingenfeld et al., 2010). Furthermore, test-retest reliability has been shown to be acceptable (*r* = .79–.86 over an average of 4 months).

**LSAS.** The Liebowitz Social Anxiety Scale (LSAS; Liebowitz, 1987) assesses social anxiety disorder. It consists of 24 items on social situations where respondents rate their level of fear and avoidance on a 4-point Likert scale for the previous week. Scores range from 0 to 144, with higher scores indicating greater social anxiety.

**RRS.** The Ruminative Response Scale (RRS; Nolen-Hoeksema and Morrow, 1991) evaluates trait rumination through 22 items rated on a 4-point Likert scale from "hardly ever" to "almost always." Scores range from 22 to 88, with higher scores indicating greater rumination.

**Qualitative Text analysis**

Both raters read the text of the participants and categorized it regarding the following predefined categories: (1) rumination on bad performance and related deficits, (2) rumination on consequences, (3) focus on negative affect, (4) reflective ruminations, (5) (cognitive) problem-solving, (6) reframing, (7) mindfulness, (8) self-compassion, (9) acceptance, (10) distraction, (11) suppression, (12) breath focus, (13) relaxation, (14) avoidance. Following the text categorization both raters rated the “strength” of the category on a scale from 0 to 4 (0 = no content, 1 = the text could maybe fall into the category, 2 = the text falls into the category but with no clear index of the strength, 3 = the text explicitly falls into the category, e.g. more than two sentences, clear signal words on the strength and duration as “quite, the whole time”, 4 = the texts falls into the category and is a model example with many sentences or strong index words, e.g. “totally, highly, completely”). Finally, raters checked if text was assigned to a category by the corresponding other rater, which was not captured in the first rating. Corresponding omissions and errors in the first rating process were then corrected if there was agreement. Interrater reliability scores can be inspected in the supplementary material table 1.

|  | Scale | Kappa (95% CI) | Weighted kappa | Correlation |
| --- | --- | --- | --- | --- |
|  |  |  |  |  |
| 1 | rumination (performance) | 0.306 [0.199; 0.413] | 0.635 | 0.867 |
| 2 | rumination (consequences) | 0.487 [0.359; 0.615] | 0.734 | 0.887 |
| 3 | negative affect | 0.528 [0.410; 0.646] | 0.761 | 0.924 |
| 4 | reflective rumination | 0.276 [0.160; 0.391] | 0.623 | 0.864 |
| 5 | (cognitive) problem-solving | 0.525 [0.382; 0.668] | 0.739 | 0.866 |
| 6 | Reframing | 0.531 [0.413; 0.648] | 0.761 | 0.883 |
| 7 | Mindfulness | 0.494 [0.319; 0.668] | 0.762 | 0.901 |
| 8 | self-compassion | 0.741 [0.614; 0.869] | 0.867 | 0.933 |
| 9 | Acceptance | 0.774 [0.582; 0.967] | 0.897 | 0.968 |
| 10 | Distraction | 0.673 [0.555; 0.792] | 0.83 | 0.926 |
| 11 | Suppression | 0.457 [0.256; 0.659] | 0.481 | 0.558 |
| 12 | breath focus | 0.575 [0.336; 0.813] | 0.617 | 0.616 |
| 13 | Relaxation | 0.587 [0.404; 0.771] | 0.754 | 0.875 |
| 14 | avoidance | 0.366 [0.226; 0.507] | 0.539 | 0.658 |

Supplementary Table 1: Interrater correlations of the qualitative scales.

**Scale Definition:**

- **Rumination on bad performance and related deficits:** Re-examination of poor performance or rumination about one's problems, worries, deficits, and symptoms.
- **Rumination on consequences:** Dwelling about negative consequences. This includes rumination on topics that are not directly associated with the TSST but have been triggered (e.g., rumination about financial worries).
- **Focus on negative affect:** Ruminative dwelling on negative affect.
- **Reflective rumination:** Distanced thoughts on the past without emotional involvement.
- **Cognitive Problem Solving:** Here, one attempts to find concrete solutions to immediate problems through thoughtful analysis. This process is characterized by a flexible consideration of possible solutions and their long-term viability. Various alternatives are evaluated, implemented, and assessed for success.
- **Reframing:** When reframing, one tries to adopt different, especially more positive perspectives. For instance, this can involve viewing new challenges as opportunities. In practical terms, reframing might also mean seeing the TSST as something unusual or amusing.
- **Mindfulness:** Mindfulness involves observing events and associated emotions in a non-judgmental manner and staying present in the moment rather than dwelling on the past or worrying about the future.
- **Self-Compassion:** Self-compassion entails maintaining a positive self-image despite difficult circumstances and appreciating oneself. This means not reacting with self-contempt or self-reproach. Key phrases include "pride" and "well done."
- **Acceptance:** Acceptance involves acknowledging the unchangeable aspects of life and incorporating them into a positive life context. It's important to note that acceptance should not be confused with resignation. Saying, "It's just the way it is, I can't change it," does not signify acceptance. Instead, acceptance means integrating unchangeable negative aspects of life into a positive life perspective.
- **Distracting:** Distracting involves focusing on other activities, such as work, watching TV, or engaging in something else to divert attention from one's emotions. It's important to distinguish distracting from normal "mind-wandering" and assess its effectiveness in Step 2. Distracting involves intentionally redirecting one's thoughts away from their emotions, not passively allowing thoughts to wander.
- **Suppression:** By suppressing emotions, one attempts to regain control or maintain emotional composure. This may involve suppressing emotions and thoughts in various ways but always aims to avoid feeling those emotions or thinking those thoughts.
- **Breath Regulation:** A body-centered regulation technique that focuses on controlling one's breath.
- **Relaxation:** A body-focused regulation method that involves relaxing the muscles.
- **Avoidance:** Expressing a desire to discontinue participation in the study or expressing concerns about making the wrong decision by participating.

| Fixed Effects | lIFG | lDLPFC | rIFG | rDLPFC | SAC |
| --- | --- | --- | --- | --- | --- |
| Constant | 0.32  (0.21) | 0.14  (0.20) | -0.09  (0.21) | -0.05  (0.21) | 0.31  (0.18) |
| Condition | -0.27  (0.31) | -0.53#  (0.30) | -0.34  (0.32) | -0.39  (0.30) | **-0.89*****  **(0.27)** |
| Group | -0.07  (0.08) | -0.01  (0.08) | 0.02  (0.07) | 0.03  (0.08) | -0.07  (0.07) |
| Performance | -0.02  (0.03) | 0.00  (0.03) | 0.03  (0.03) | 0.04  (0.03) | 0.01  (0.02) |
| Performance^2 | 0.00  (0.00) | -0.00  (0.00) | -0.00  (0.00) | -0.00  (0.00) | -0.00  (0.00) |
| Condition*  Group | 0.11  (0.07) | **0.21****  **(0.07)** | **0.16***  **(0.07)** | **0.18***  **(0.07)** | **0.18****  **(0.06)** |
| Condition*  Performance | 0.04  (0.04) | 0.08#  (0.04) | 0.04  (0.04) | 0.06  (0.04) | **0.11****  **(0.03)** |
| Condition*  Performance^2 | -0.00  (0.00) | **-0.00***  **(0.00)** | -0.00  (0.00) | -0.00  (0.00) | **-0.00****  **(0.00)** |

Supplementary Table 2. Effects of the mixed models on fNIRS data controlling for performance #*p* < .1,**p* < .05,***p* < .01,****p* < .001

| Group 1 | Group 2 | rank | p | FDR critical p-val. | d |
| --- | --- | --- | --- | --- | --- |
| HC | MDD abuse no anxiety | 1 | 0.001 | 0.005 | 1.24 |
| HC | MDD no abuse no anxiety | 2 | 0.002 | 0.010 | 0.86 |
| HC | MDD anxiety abuse | 3 | 0.002 | 0.015 | 1.36 |
| MDD no abuse no anxiety | MDD anxiety no abuse | 4 | 0.010 | 0.020 | 0.88 |
| MDD abuse no anxiety | MDD anxiety no abuse | 5 | 0.012 | 0.025 | 1.15 |
| MDD anxiety no abuse | MDD anxiety abuse | 6 | 0.024 | 0.030 | 1.18 |
| MDD no abuse no anxiety | MDD abuse no anxiety | 7 | 0.223 | 0.035 | 0.27 |
| HC | MDD anxiety no abuse | 8 | 0.290 | 0.040 | 0.17 |
| MDD no abuse no anxiety | MDD anxiety abuse | 9 | 0.370 | 0.045 | 0.41 |
| MDD abuse no anxiety | MDD anxiety abuse | 10 | 0.740 | 0.050 | 0.16 |

Supplementary Table 3. FDR-corrected post-hoc tests of the exploratory comparison.

Supplementary Tables 4a-c: Subgroups of the exploratory analysis

|  | | MDD Group | | N |
| --- | --- | --- | --- | --- |
|  |  | HC | MDD |  |
| Emotional abuse | No | 39 | 35 | 74 |
|  | Yes | 3 | 18 | 21 |
| N | | 42 | 53 | 95 |

|  | | MDD Group | | N |
| --- | --- | --- | --- | --- |
|  |  | HC | MDD |  |
| Social Anxiety | No | 42 | 35 | 77 |
|  | Yes | 0 | 19 | 19 |
| N | | 42 | 54 | 96 |

|  | | MDD Group | | N |
| --- | --- | --- | --- | --- |
|  |  | HC | MDD |  |
| Exploratory Analysis Subgroups | HC | 39 | 0 | 39 |
|  | MDD no abuse no anxiety | 0 | 23 | 23 |
|  | MDD abuse no anxiety | 0 | 11 | 11 |
|  | MDD anxiety no abuse | 0 | 12 | 12 |
|  | MDD anxiety abuse | 0 | 7 | 7 |
| N | | 39 | 53 | 92 |


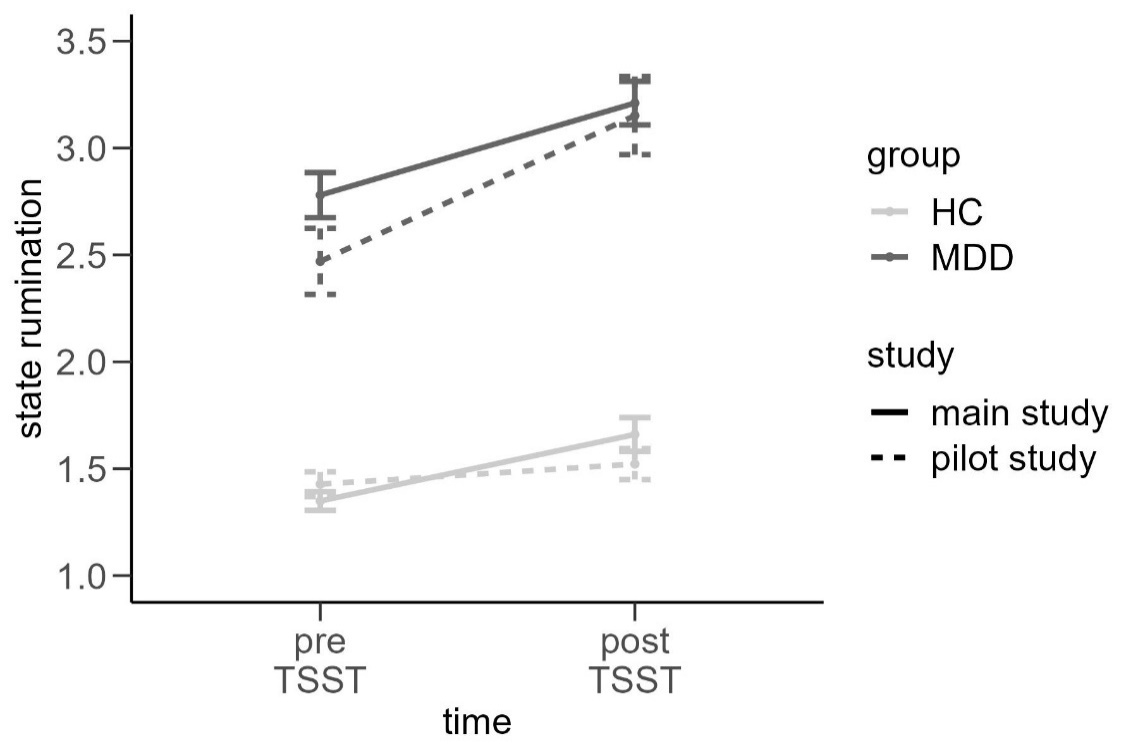


Supplementary figure 1: comparison of baseline state rumination and -reactivity from the actual study and our previous study (Rosenbaum et al. 2021)


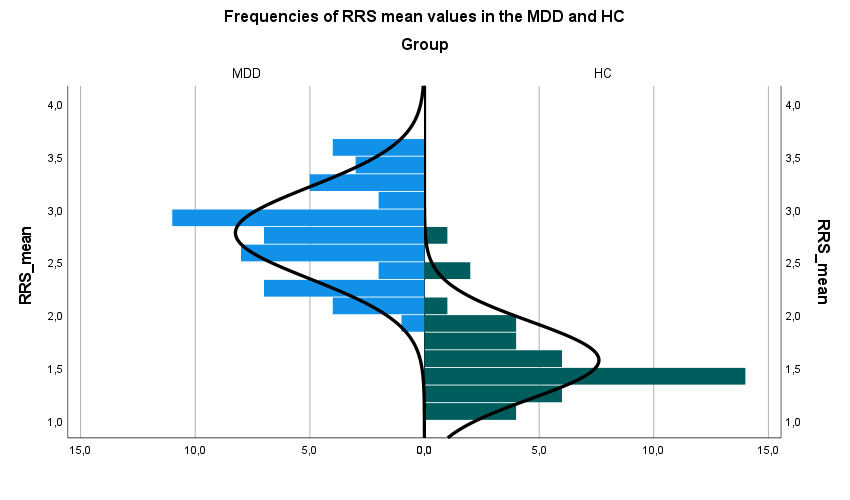


Supplementary Figure 2: Frequencies of the RRS distribution within the HC and MDD sample.

| **Supplementary Table 5: Frequencies of RCI within the clinical groups** | | | | | | |
| --- | --- | --- | --- | --- | --- | --- |
|  | | | RCI | | | Total |
|  |  |  | No Change | Reliable Decrease | Reliable Increase |  |
| Group | HC | Frequency | 35 | 0 | 7 | 42 |
|  |  | % Group | 83,3% | 0,0% | 16,7% | 100,0% |
|  |  | % RCI | 50,0% | 0,0% | 30,4% | 43,3% |
|  | MDD | Frequency | 35 | 4 | 16 | 55 |
|  |  | % Group | 63,6% | 7,3% | 29,1% | 100,0% |
|  |  | % RCI | 50,0% | 100,0% | 69,6% | 56,7% |
| Total | | Frequency | 70 | 4 | 23 | 97 |
|  |  | % Group | 72,2% | 4,1% | 23,7% | 100,0% |
|  |  | % RCI | 100,0% | 100,0% | 100,0% | 100,0% |

Analysis of the RCI of state Rumination increases within the Patient Groups. RCI was computed with $RCI= \frac{y_{1,i}-y_{0,i}}{s_{Diff}}$ with $S_{Diff}= \sqrt{2*(S_{0}\sqrt{1-r_{yy})}}^{2}$ with $S_{0}$ as the Standard Deviation of the state rumination questionnaire pre TSST ($S_{0}=.94$) and $r_{yy}$ as the retest reliability ($r_{yy}=.92)$. The distribution is different between HC and MDD χ²(2)=7.415, p<.05

| **Supplementary Table 6: Frequencies of RCI within the clinical subgroups** | | | | | | | |
| --- | --- | --- | --- | --- | --- | --- | --- |
|  | | | | RCI | | | Total |
|  |  |  |  | No Change | Reliable Decrease | No Change |  |
| Groups | HC | Frequencies | | 33 | 0 | 6 | 39 |
|  |  | % Group | | 84,6% | 0,0% | 15,4% | 100,0% |
|  |  | % RCI | | 50,0% | 0,0% | 27,3% | 42,4% |
|  | MDD no abuse no anxiety | Frequencies | | 16 | 3 | 4 | 23 |
|  |  | % Group | | 69,6% | 13,0% | 17,4% | 100,0% |
|  |  | % RCI | | 24,2% | 75,0% | 18,2% | 25,0% |
|  | MDD abuse no anxiety | Frequencies | | 5 | 0 | 6 | 11 |
|  |  | % Group | | 45,5% | 0,0% | 54,5% | 100,0% |
|  |  | % RCI | | 7,6% | 0,0% | 27,3% | 12,0% |
|  | MDD anxiety no abuse | Frequencies | | 8 | 1 | 3 | 12 |
|  |  | % Group | | 66,7% | 8,3% | 25,0% | 100,0% |
|  |  | % RCI | | 12,1% | 25,0% | 13,6% | 13,0% |
|  | MDD anxiety abuse | Frequencies | | 4 | 0 | 3 | 7 |
|  |  | % Group | | 57,1% | 0,0% | 42,9% | 100,0% |
|  |  | % RCI | | 6,1% | 0,0% | 13,6% | 7,6% |
| Total | | | Frequencies | 66 | 4 | 22 | 92 |
|  |  |  | % Group | 71,7% | 4,3% | 23,9% | 100,0% |
|  |  |  | % RCI | 100,0% | 100,0% | 100,0% | 100,0% |

Analysis of the RCI of state Rumination increases within the Patient Subgroups. The distribution is uneven χ²(8)=16.069, p<.05

**Supplementary Table 7. Exploratory Regression Analysis of state rumination reactivity including all predictors.**

|  | Unstandardized Beta | Coefficients Std.-Error |  | Beta-value | t-value | p-value |
| --- | --- | --- | --- | --- | --- | --- |
| Intercept | 0.050 | 0.346 |  |  | 0.145 | 0.885 |
| LSAS | 0.012 | 0.004 |  | 0.452 | 2.993 | 0.004 |
| CTQ Emotional Abuse | 0.056 | 0.022 |  | 0.396 | 2.507 | 0.014 |
| CTQ Emotional Neglect | -0.007 | 0.022 |  | -0.048 | -0.308 | 0.759 |
| CTQ Physical Abuse | 0.019 | 0.038 |  | 0.068 | 0.496 | 0.621 |
| CTQ Physical Neglect | -0.039 | 0.034 |  | -0.139 | -1.129 | 0.262 |
| CTQ Sexual Abuse | -0.009 | 0.033 |  | -0.037 | -0.286 | 0.776 |
| Number of depressive Episodes | -0.094 | 0.060 |  | -0.244 | -1.561 | 0.122 |
| RRS | -0.022 | 0.186 |  | -0.024 | -0.120 | 0.904 |
| BDI | -0.011 | 0.011 |  | -0.203 | -0.985 | 0.328 |
